# Inequities in curative health-care utilization among the adult population (20–59 years) in India: A comparative analysis of NSS 71^st^ (2014) and 75^th^ (2017–18) rounds

**DOI:** 10.1371/journal.pone.0241994

**Published:** 2020-11-25

**Authors:** Shreya Banerjee, Indrani Roy Chowdhury

**Affiliations:** Centre for the Study of Regional Development, School of Social Sciences, Jawaharlal Nehru University, New Delhi, India; University of West London, UNITED KINGDOM

## Abstract

**Objective:**

The study attempts (a) to compute the degree of socio-economic inequity in health care utilization and (b) to decompose and analyze the drivers of socio-economic inequity in health care utilization among adults (20–59 years) in India during the periods 2014 and 2017–18.

**Data source:**

The analysis has been done by using the unit level data of Social Consumption: Health (Schedule number 25.0), of National sample Survey (NSS), corresponding to the 71st and 75^th^ rounds.

**Methods:**

Odds ratios were computed through logistic regression analysis to examine the effect of the socio-economic status on the health seeking behaviour of the ailing adult population in India. Concentration Indices (CIs) were calculated to quantify the magnitude of socio-economic inequity in health care utilization. Further, the CIs were decomposed to find out the share of the major contributory factors in the overall inequity.

**Results:**

The regression results revealed that socio-economic status continues to show a strong association with treatment seeking behavior among the adults in India. The positive estimates of CIs across both the rounds of NSS suggested that health care utilization among the adults continues to be concentrated within the higher socio-economic status, although the magnitude of inequity in health care utilization has shrunk from 0.0336 in 2014 to 0.0230 in 2017–18. However, the relative contribution of poor economic status to the overall explained inequities in health care utilisation observed a rise in its share from 31% in 2014 to 45% in 2017–18.

**Conclusion:**

To reduce inequities in health care utilization, policies should address issues related to both supply and demand sides. Revamping the public health infrastructure is the foremost necessary condition from the supply side to ensure equitable health care access to the poor. Therefore, it is warranted that India ramps up investments and raises the budgetary allocation in the health care infrastructure and human resources, much beyond the current spending of 1.28% of its GDP as public expenditure on health. Further, to reduce the existing socio-economic inequities from the demand side, there is an urgent need to strengthen the redistributive mechanisms by tightening the various social security networks through efficient targeting and broadening the outreach capacity to the vulnerable and marginalized sections of the population.

## Introduction

Health is a “critically significant constituent of human capabilities”, i.e. an integral enabling factor for a person to thrive as a human being and health equity is central to social justice [1]. That is why the inequalities in health and health care have been flagged as more critical than inequalities in most other domains [1, 2]. Since the decade of 1990s, policy oriented research in health inequities has emerged as an increasingly popular domain of investigation worldwide with national governments, policy-makers and international organizations like the World Bank and the World Health Organization starting to put equity among the top priorities in their agendas [3–5]. This is reflected in the *Millennium Development Goals* (Goals- 4, 5 and 6) that laid primary thrust on the improvement in the health outcomes of the world’s poor and subsequently in the Goal-3 of *Sustainable Development Goals* that also focused on ensuring equity in health [6, 7]. Studies have testified that the inequalities in health outcomes and access to health care are due to the differences in economic constraints between the poor and the rich rather than the differences in their preferences [8–12]. Such inequalities should not be considered simply as inequalities but as inequities as they are socially unjust [13]. The report of the *Commission on Social Determinants of Health (CSDH)*, set up by the World Health Organization (WHO) in 2005, also observed that rather than differences in individual behaviors, it is a “cluster of random events” that systematically keeps the health of some population groups worse than others. Inadequate and inefficient health care delivery system were flagged as important determinants of health outcomes [14]. India’s National Health Policy in 2002 has recognized *‘ensuring a more equitable access to health services across the social and geographical expanse of the country’*, as an important objective [15]. Moreover, the development in health inequity literature [16–19] has motivated researchers in India to estimate the inequities in health status and distribution of health care services across the various socio-economic groups [20–25].

Ideally, access to health care is a human right that all citizens of a country irrespective of their socio-economic position should rightfully enjoy. However, in a developing country like India, with 29.5 per cent of its population living under the poverty line (fixed at 32 INR per day for rural areas and 47 INR for urban areas) during 2011–12 [26], health is a privilege for a large section of its population. The latest round of *National Sample Survey on Social Consumption*: *Health* [27], shows that the proportion of persons reported as ailing (PPRA) is unevenly distributed across the economic groups with the poorest reporting the lowest PPRA of 3.79 compared to that of the richest (8.17). The rate of treatment seeking is also higher among the rich (93.5%) compared to the poor (86.3%). This is indicative of higher tolerance and lack of awareness among the poor people for the mortality and morbidity risks. This higher tolerance is also reflective of the fact that treatment seeking of the poor is often associated with high opportunity costs of wage loss, which the poor find difficult to afford. The poor often thus indulge in self-treatment to save time and money [27]. The skewed time preference of the poor, in terms of discounting heavily the future health risks, induce them to postpone treatment for minor illnesses and seek treatment when in the verge of collapse and thereby ending up incurring exorbitantly high out of pocket expenditures, which often become catastrophic [28–32].

In terms of efficiency in health care deliveries, two aspects are critically important–accessibility and utilization. While the former covers the supply side, the latter broadly covers the demand side. According to the *National Health Systems Resource Centre (NHSRC) Report 2017* [33], 70 percent of the aggregate health care expenditure are incurred through out of pocket (OOP) expenditure and 75 percent of the morbidities are treated through private sectors at exorbitant costs. Under this backdrop, the NSS Social Consumption: Health (2019) [27] data reveals that the bottom two income-quartiles accounted for almost 63% of the total unmet need for health care. The inequality in unmet need for health care was far worse in rural sector with 78% of the share borne by the two lowest income-quartiles. Moreover, only 39.09% of ailing persons belonging to the poorest quartile availed treatment in a government hospital, while 25% of the richest quartile availed services of a government facility. Majority of the ailing poor have to resort to the services of the private hospital or clinics (56%), which are quicker in service-delivery. In India, the lowest wealth quintile of population is reported to utilize only one-tenth of public health subsidy as opposed to nearly one-third by the richest wealth quintile [34]. This inequality is inequitable as it arises out of a person’s marginalization in the society.

While research in the domain of inequity in health care utilization has gained momentum in India since mid-2000, the studies dedicated to this issue have been largely limited to the maternal health care utilization [35–40] or child immunization, i.e. preventive health care utilization [41–45]. The studies that have investigated the inequities in health care utilization in India have methodological limitations as they are mostly restricted to only bivariate analysis [46–49]. As opposed to the developed countries, there is, by and large, very thin literature in India that examine the issues of socio-economic inequity in health care utilization by employing the newer econometric tools of measurement of health inequality [20, 22–25, 50] and most of these existing studies have either done an aggregated analysis for all ages or have focused only on the elderly population. The issue of inequity in health care utilization among adults remains under-researched in India. The aim of the present study, is, therefore, to plug this research gap.

Given that the age dependency ratio in India is 49.25% (i.e., 39.73% and 9.52 for young and old population respectively) [51], India has a considerably large proportion of the adult population (47.2%) between the ages of 20 and 59 years [52]. Healthy adulthood is the bedrock of a country’s human resource base. This age-group forms the workforce and acts as engines of economic growth. It is therefore, imperative that the working age-group stays healthy and has equitable access to health care services in case of illnesses. Since, an overwhelming majority of the workforce in India (82%) is employed in the unorganized sector [53] characterized by unregulated and unsafe working conditions, they are highly vulnerable to various disease burdens as well as workplace-related health hazards. Unintentional injuries constitute the largest share of deaths (roughly 26%) among the younger adult age-groups. Among the middle-aged adults, however, the leading causes of death are cardiovascular diseases, accounting for roughly 32% deaths [54]. However, due to inequitable access to health care, the disadvantaged groups even succumb to easily curable diseases, in the absence of medical attention. Given the huge disparity in health indicators among the wealth quintiles [24, 55], a study of inequity in health care utilization is extremely warranted for a targeted policy intervention.

Given this backdrop, the central concern of the study is, therefore, to delve into the complex interplay of a person’s health seeking behavior and their position in the socio-economic gradient. The primary objectives of the study are: (a) computing the degree of socio-economic inequity in utilization of health care and (b) observing its prominent contributory factors among the adults in India (20–44 years or younger adults and 45–59 years middle-aged). We also aim to see if there have been any changes in inequity and its drivers between the two NSS rounds, i.e. 71^st^ (2014) and 75^th^ (2017–18).

## Materials and methods

### Data source

The analysis has been done by using the unit level randomized data collected through 71st and 75^th^ rounds of the National sample Survey (NSS) [27, 56], corresponding to the Schedule number 25.0 (Social Consumption: Health), collected during the period of January—June 2014 and July 2017 –June 2018 respectively. The NSS 71^st^ and 75^th^ rounds survey data provide a nationally representative detailed information on self-reported morbidity episodes and the corresponding treatment seeking for a specified recall period along with a comprehensive background information of that particular individual’s socio-economic and demographic characteristics. The socio-economic and demographic profile of our samples in the age-group 20–59 years, pertaining to both the 71^st^ and 75^th^ rounds is presented in Table 1. It must be noted here that adults are not a homogenous group and display a great degree of heterogeneity. For the present study, this broad age-group is bifurcated into younger adults aged between 20 and 44 years and middle-aged persons aged 45–59 years.

**Table 1:** Socio-economic and demographic profile of the sample.

Background Characteristics		Adult Population (20 to 59 years)*			
		2014 (71^st^ round)		2017–18 (75^th^ round)	
		Frequency	(%)	Frequency	(%)
Age	Younger Adults (20–44 years)	132,733	73.68	222,899	70.94
	Middle Aged (45–59 years)	47,411	26.32	91,304	29.06
Sex	Male	89,454	49.66	159,495	50.762
	Female	90,690	50.34	154,697	49.235
	Transgender	-	-	11	0.004
Place of Residence	Rural	99,148	55.04	215,838	68.69
	Urban	80,996	44.96	98,365	31.31
Education	Illiterate	40,249	22.45	76,364	24.30
	Upper Primary or below	67,687	37.76	109,449	34.83
	Secondary	24,669	13.76	46,092	14.67
	Higher secondary or above	46,646	26.02	82,298	26.19
Marital Status	Currently Married	144,391	80.55	250,653	79.77
	Others	34,860	19.45	63,540	20.23
Religion	Hindu	137,885	76.54	258,071	82.14
	Muslim	25,169	13.97	40,438	12.87
	Christian	10,616	5.89	7,331	2.33
	Sikh	3,280	1.82	5,687	1.81
	Others	3,194	1.77	2,676	0.85
Social Group	ST	23,135	12.84	28,191	8.97
	SC	29,467	16.36	60,325	19.20
	OBC	70,915	39.37	139,802	44.49
	Others	56,627	31.43	85,885	27.33
Economic Group	Poor	47,963	26.63	63,327	20.15
	Lower Middle	35,849	19.90	74,471	23.70
	Upper Middle	51,149	28.40	82,197	26.16
	Rich	45,158	25.07	94,208	29.98
Health Coverage	Not Covered	149,694	83.51	261,454	83.21
	Covered	29,555	16.49	52,745	16.79
Household Size	4 or less	59,382	32.96	140,418	44.69
	5 to 6	62,440	34.66	108,203	34.44
	7 or more	58,322	32.38	65,582	20.87
Region	North	27,028	15.00	41,969	13.36
	Central	38,091	21.14	75,438	24.01
	East	31,946	17.73	65,954	20.99
	Northeast	22,875	12.70	12,796	4.07
	West	24,864	13.80	46,022	14.65
	South	35,340	19.62	72,024	22.92
**TOTAL**		**179,251**	**100**	**314,203**	**100**

Note

* unweighted sample

Source: Authors’ calculation based on the NSS 71^st^ and 75^th^ Round data on Social Consumption: Health

In this study, out of the total sample size, only those adults (20–59 years) who self-reported some illness in the past 15 days recall period were considered for analyses related to health care utilization. Health care facilities are supposed to be utilized only by those who need it, i.e. those who suffer from any ailment that require medical treatment. Thus, the number of observations for our analyses pertaining to utilization of health care (inclusive of both in-patient and out-patient treatments) was restricted to 18,445 (9244 and 9201 for younger adults and middle-aged respectively) for 71^st^ round and 20,157 (8680 and 11,447 for younger adults and middle-aged respectively) for 75^th^ round. It is to be noted that the figures do not pertain to number of ailing persons, rather number of spells of ailment reported. This means that there may be more than one spell of ailment reported by a single individual which has been reported as separate observations because treatment must be sought for each incidence of illness by an individual. The number of individuals who reported multiple spells of illnesses was 1346 (430 and 916 for younger adults and middle-aged respectively) for 71^st^ round and 1132 (243 and 889 for younger adults and middle-aged respectively) for 75^th^ round. Ailments reported among the younger adults (20–44 years) were majorly related to infection and respiratory in both the 71^st^ and the 75^th^ rounds. The share of infection related ailments increased from 24 to 31.5 percent between the two rounds. In case of the middle-aged population (45–59 years), diabetes and hypertension were the most commonly reported ailments across the time periods 2014 and 2017–18. The frequency distributions of number of spells of illnesses and the nature of illnesses have been presented in Tables 2 and 3.

**Table 2:** Frequency distribution of individuals reporting one or multiple illnesses among adult population in India.

	2014 (71st round)						2017–18 (75th round)					
Number of Spells	Younger Adults (20–44 years)		Middle-Aged (45–59 years)		All Adults (20–59 years)		Younger Adults (20–44 years)		Middle-Aged (45–59 years)		All Adults (20–59 years)	
	Frequency	%	Frequency	%	Frequency	%	Frequency	%	Frequency	%	Frequency	%
1	8338	95.10	7124	88.6	15462	91.99	8170	97.11	9,501	91.44	17,671	93.98
2	390	4.45	723	9.0	1113	6.62	225	2.67	727	7.00	952	5.06
3	36	0.41	150	1.9	186	1.11	13	0.15	131	1.26	144	0.77
4	3	0.03	34	0.4	37	0.22	4	0.05	26	0.25	30	0.16
5	0	0.00	9	0.1	9	0.05	1	0.01	5	0.05	6	0.03
6	1	0.01	0	0.0	1	0.01	0	0	0	0	0	0
Total	8768	100	8040	100	16808	100	8413	100	10390	100	18803	100

Source: Authors’ calculation based on the NSS 71^st^ and 75^th^ rounds’ data on Social Consumption: Health

**Table 3:** Nature of ailments reported by the adult population in India.

Nature of Ailment	2014 (71st round)						2017–18 (75th round)					
	Younger Adults (20–44 years)		Middle-Aged (45–59 years)		All Adults (20–59 years)		Younger Adults (20–44 years)		Middle-Aged (45–59 years)		All Adults (20–59 years)	
	Frequency	%	Frequency	%	Frequency	%	Frequency	%	Frequency	%	Frequency	%
Infection	2,250	24.34	988	10.74	3,238	17.55	2,739	31.56	1,395	12.15	4,134	20.51
Cancer	88	0.95	99	1.08	187	1.01	77	0.89	178	1.55	255	1.27
Blood diseases	137	1.48	87	0.95	224	1.21	108	1.24	105	0.91	213	1.06
Diabetes	456	4.93	1665	18.1	2121	11.5	574	6.61	2626	22.88	3200	15.88
Endocrine, metabolic, nutritional	290	3.14	224	2.43	514	2.78	373	4.3	352	3.07	725	3.6
Psychiatric and Neurological	814	8.81	531	5.77	1,345	7.29	633	7.29	559	4.87	1,192	5.91
Eye	108	1.17	125	1.36	233	1.26	81	0.93	127	1.11	208	1.03
Ear	56	0.61	43	0.47	99	0.54	38	0.44	38	0.33	76	0.38
Hypertension	467	5.05	1497	16.27	1964	10.65	530	6.11	2339	20.9	2929	14.53
Cardio-Vascular	234	2.53	507	5.51	741	4.02	234	2.7	649	5.65	883	4.38
Respiratory	1,171	12.67	872	9.48	2,043	11.08	888	10.23	705	6.14	1,593	7.9
Gastro-intestinal	914	9.89	576	6.26	1,490	8.08	691	7.96	512	4.46	1,203	5.97
Skin	225	2.43	142	1.54	367	1.99	241	2.78	164	1.43	405	2.01
Musculo-Skeletal	821	8.88	1,237	13.44	2,058	11.16	558	6.43	1,197	10.43	1,755	8.71
Genito-urinary	380	4.11	170	1.85	550	2.98	272	3.13	163	1.42	435	2.16
Obstetric	239	2.59	1	0.01	240	1.3	201	2.32	1	0.01	202	1
Injuries	347	3.75	196	2.13	543	2.94	274	3.16	170	1.48	444	2.2
Others	247	2.67	241	2.62	488	2.65	168	1.94	137	1.19	305	1.51
Total	9,244	100	9,201	100	18,445	100	8,680	100	11,477	100	20,157	100

Source: Authors’ calculation based on the NSS 71^st^ and 75^th^ rounds’ data on Social Consumption: Health

### Variables for statistical analyses

#### Dependent variable

“Whether any treatment was taken on medical advice for a reported spell of ailment” (Yes = 1, No = 0) has been chosen as the dependent variable. In both the 71^st^ and 75^th^ rounds of NSS, information was collected on the nature of treatment sought for the spells of ailment during the last 15 days recall period. This includes treatment for ailments that started within the past 15 days as well as those that started more than 15 days ago but were ongoing during the recall period; the ailments may be acute or chronic.

In this study, all the categories of treatment except no treatment were clubbed together, thereby getting the binary category- medical treatment sought (1), no medical treatment sought (0). Further, the cases of reported self-treatment were re-categorized in the no medical treatment category.

#### Predictor variables

Four broad domains of covariates have been identified that may induce inequalities in health care utilization. These domains pertain to sets of demographic factors, socio-economic factors, institutional factors and geographical factors.

The demographic variables include: (a) age (categorized as younger adults (20–44 years) and middle aged (45–59 years), due to the varying disease burden and health seeking behavior of the two age cohorts); (b) sex (male and female); (c) social groups (Scheduled Castes (SC), Scheduled Tribes (ST), Other Backward Classes (OBC) and others); (d) religion (Hindu, Muslim, Christian, Sikh, and others and (e) marital status (currently married and others (inclusive of those who never married or are divorced/ separated/ widowed)).

The socio-economic variables comprise of (a) economic group: given that NSS provides data on households’ monthly consumption expenditure (MPCE) and not income, we have taken MPCE as a proxy for income. MPCE quartiles were created based on the relative ranking of the households as per standard of living (NSSO 2019); (b) educational status: the NSS collected information on the general educational level of individuals in 15 categories that were contracted into four broad classifications vis a vis illiterate; upper primary or below; secondary; higher secondary and above for the present study; (c) household size (categorized as 4 or less; 5 to 6; and 7 or more).

The institutional variable comprises of health insurance (categorized as covered and not covered). Lastly, the geographical variables include (a) place of residence (urban and rural); and (b) region (North, East, West, Central, Northeast and South) to account for the effect of the regional imbalance in India.

### Statistical analyses

Bivariate percentage distribution (cross-tabulation) is calculated to estimate the differences in PAP (Proportion of Ailing Persons per 100) and percentage of ailing persons seeking treatment on medical advice by predictor variables. The results are tested for statistical significance by using Pearson’s Chi-squared test for homogeneity or independence.

The association between predictor variables and health seeking behavior is examined by considering binary logistic model. The equation is presented as follows:

$$Pi=pr\left(y=1|x\right)=F\left(x\beta \right)=\frac{\exp\left(x\beta \right)}{1+\exp\left(x\beta \right)}$$ (1)

*F*(*xβ*) follows the cumulative distribution function of the logistic distribution. Eq (1) is not linear in parameter and requires certain manipulations to get a logistic function, L which is linear in parameter.

$$L=\mathrm{Log}\left(\frac{p}{1-p}\right)=x_{i}\beta +u$$ (2)

L gives the log of odds in favor of *y*_i_ = 1/*x*_i_, where *x*_i_ is the vector of socio-economic covariates of *i*^*th*^ individual, the coefficients βare parameters to be estimated, and *u* is the idiosyncratic error term.

A number of studies have estimated the level of inequalities in health, using various techniques like Lorenz curve, Gini coefficient, Concentration Index and Concentration Curve [13, 17, 19, 45, 57–61]. In the present study, Concentration Index has been chosen to measure the magnitude of socio-economic inequity in health care utilization as it is the best suited for our objective. This is because, by computing Gini coefficient one can only measure pure health inequality and not socio-economic inequality in health [62]. Gini coefficient (based on the Lorenz curve) is computed by ranking individuals to their health status whereas in the computation of the Concentration Index (based on the Concentration Curve), individuals are ranked according to their socio-economic status [63, 64].

Following Wagstaff (2005) [65], the magnitude of socio-economic inequity in utilization of health care was quantified through Concentration Index (CI), by using the equation:

$$CI=\frac{1}{n}\overset{n}{\underset{i=1}{\sum }}\left[\frac{\left(a-b\right)}{\left(a-\mu \right)\left(\mu -b\right)}\left(2r_{i}-1\right)\right]$$ (3)

Where,

n = sample size

μ = weighted mean of the health variable of the sample

r_i_ = fractional rank of the i^th^ individual (for weighted data) in terms of their household’s economic status. Monthly Per-capita Consumption Expenditure- MPCE (obtained by dividing monthly usual consumption expenditure of a household by the size of the household), is the rank variable for this analysis.

a = 1, and b = 0 are the maximum and minimum levels of health care utilization respectively.

The value of CI varies between −1 and +1. A negative value implies that the outcome of the variable is concentrated among socio-economically disadvantaged people while a positive value means the inequity is pro-rich.

Further, the concentration indices are decomposed to find out the share of the major contributory factors. The contribution of each of the predictors is computed as a percentage of the total inequality in health care utilization. For any additive linear regression model, association of a health variable, y, to a set of k_i_ determinants- X_ki_ can be expressed as follows [18]:

$$y_{i}=\alpha +\sum \beta_{k}x_{k}+\varepsilon$$ (4)

Given the association between y_i_ and x_k_ in Eq (4), the concentration index for y_i_ health variable (C) can be expressed as follows [18]:

$$C=\sum \left(\frac{\beta_{k}X_{k}}{\mu }\right)C_{k}+\frac{GC_{e}}{\mu }$$ (5)

Where,

X_k_ is the mean of the x_k_ determinant,

C_k_ is the concentration index of the x_k_ determinant

μ is the mean of the health outcome

GCε/ μ is the generalized concentration index for the error term or the residual component (unexplained inequity in health outcome)

The absolute contribution of each determinant is the product of the sensitivity (elasticity) of health variable with respect to that determinant and the degree of MPCE-based inequality in that determinant (concentration index, C_k_) which is expressed as (β_k_ X_k_ /μ) * C_k_. The percentage contribution of each determinant is obtained by dividing its absolute contribution by C of the health variable, multiplied by 100, which is expressed as [(β_k_X_k_/μ) * (C_k_/C)] * 100 [18].

All the statistical analyses were conducted using the software STATA version 14.

## Results

### Socio-economic differentials in PAP (Proportion of Ailing Persons) and utilization of health care

Socio-economic and demographic differentials of the PAP and health care utilisation of the adult population in India are presented in Table 4. This analysis is important as it compares the morbidity rate and rate of seeking treatment by those who self-report any episode of morbidity. The results show that the proportion of (living) persons (per 100) reporting ailments at any time during past 15-days recall period has declined from 9.39 in 71^st^ round to 5.98 in 75^th^ round. Also, rate of seeking treatment on medical advice has improved from 87.42% in 71^st^ round to 90.80% in 75^th^ round. This fall in PAP and improvement in treatment seeking rate has been observed across all the covariates.

**Table 4:** Socio-economic differentials in morbidity prevalence rate and utilization of health care in adult population in India.

Covariates		2014 (71^st^ round)		2017–18 (75^th^ round)		2014 (71^st^ round)		2017–18 (75^th^ round)	
		Proportion of Ailing Persons	chi squared	Proportion of Ailing Persons	chi squared	Rate of seeking treatment (%)	chi squared	Rate of seeking treatment (%)	chi squared
		(%)		(%)					
Age	Younger Adults (20–44 years)	6.64	4500 Ϯ	3.71	7400 Ϯ	84.84	111.48 Ϯ	87.89	154.622 Ϯ
	Middle Aged (45–59 years)	17.14		11.83		90.00		93.00	
Sex	Male	7.84	494.166 Ϯ	5.10	430.456 Ϯ	87.42	0.0001	90.06	9.583 Ϯ
	Female	10.91		6.86		87.42		91.34	
Place of Residence	Rural	8.61	158.099 Ϯ	5.35	298.891 Ϯ	85.51	59.134 Ϯ	89.35	52.471 Ϯ
	Urban	10.35		6.83		89.27		92.30	
Education	Illiterate	11.70	684.207 Ϯ	8.38	1200 Ϯ	83.62	107.737 Ϯ	89.39	17.755 Ϯ
	Upper Primary or below	10.15		6.52		87.91		91.17	
	Secondary	8.33		5.06		89.25		91.74	
	Higher secondary or above	6.85		4.38		90.67		91.25	
Marital Status	Currently Married		96.455 Ϯ	6.10	33.168 Ϯ	87.52		91.05	6.957 Ϯ
		9.72					0.9008		
	Others	8.01		5.48		86.90		89.63	
Religion	Hindu	9.40	236.076 Ϯ	5.88	336.819 Ϯ	87.06	35.191 Ϯ	90.65	23.020 Ϯ
	Muslim	9.92		7.30		88.73		91.95	
	Christian	6.81		4.15		85.15		87.55	
	Sikh	15.14		7.92		94.64		93.45	
	Others	7.20		4.37		85.95		90.75	
Social Group	ST	5.46	479.536 Ϯ	3.06	837.346 Ϯ	77.25	153.223 Ϯ	81.74	158.729 Ϯ
	SC	9.90		6.18		85.80		90.21	
	OBC	9.95		6.06		88.76		91.03	
	Others	10.03		7.07		88.70		92.52	
Economic Group	Poor	6.62	1300 Ϯ	3.79	1500 Ϯ	80.42	236.972 Ϯ	86.30	173.903 Ϯ
	Lower Middle	7.80		4.91		83.61		89.66	
	Upper Middle	9.73		6.13		87.25		92.29	
	Rich	13.20		8.17		90.98		93.50	
Health Coverage	Not Covered	8.47	901.716 Ϯ	5.29	1200 Ϯ	87.32	0.369	90.11	28.185 Ϯ
	Covered	14.05		9.06		87.66		92.49	
Household Size	4 or less	12.05	922.007 Ϯ	7.76	1000 Ϯ	87.08	4.318	91.04	1.243
	5 to 6	9.21		5.43		87.23		90.65	
	7 or more	6.88		4.51		88.34		90.52	
Region	North	8.17	3600 Ϯ	6.07	2400 Ϯ	92.85	287.097 Ϯ	91.43	224.003 Ϯ
	Central	7.58		5.19		88.17		88.99	
	East	9.97		7.00		80.99		88.06	
	Northeast	2.64		1.65		75.98		79.33	
	West	8.65		5.98		86.59		93.44	
	South	16.64		8.66		89.41		93.24	
**TOTAL**	** **	**9.39**		**5.98**	** **	**87.42**		**90.80**	

Note:

Ϯ p<0.001

Source: Authors’ calculation based on the NSS 71^st^ and 75^th^ rounds’ data on Social Consumption: Health

Our analysis shows that a high rate of self-reported morbidity may not always induce a commensurate higher rate of treatment seeking by individuals belonging to various socio-economic and demographic categories. For example, with respect to the level of education, illiterates reported a higher PAP (11.7 in 71^st^ round and 8.38 in 75^th^ round) than those with some education. However, illiterates have a lower rate of seeking treatment compared to the educated population (83.62% and 89.39% in 71^st^ and 75^th^ rounds respectively).

With respect to gender, more adult females reported morbidity compared to adult men in both the rounds, but the proportion seeking treatment for an ailment was equal for both the sexes in 71^st^ round and slightly higher for females in 75^th^ round. Also, the proportion of ailing adults is higher in urban areas compared to rural areas in both the rounds. The rate of treatment seeking is also higher in urban adult population than their rural counterparts in both the rounds. Marital status also has considerable influence on the health-seeking behaviour. Those who reported being currently married had better health-seeking tendencies when compared to those who never married or are divorced/ widowed/ separated.

The rate of seeking treatment by economic status suggests that those adult individuals belonging to the richer MPCE quartiles have higher self-reported morbidity and a higher rate of treatment seeking compared to adults belonging to poorer MPCE quartile. Those who are covered by certain health insurance scheme have a marginally higher rate of treatment seeking. SCs and STs have lower rates of treatment seeking compared to OBCs and others. Household size was not a significant determinant of differences in the rate of seeking medical treatment of the adult population.

The socio-economic differentials of the ailing adult population with unmet need for health care has been presented in Table 5. Majority of the adult population, whose need for medical treatment remained unmet on experiencing a spell of ailment, belonged to the younger adults age-group (20–44 years), female sex, Hindu religion and Other Backward Castes across both the rounds- 71^st^ and 75^th^. Furthermore, more than 70% of the ailing adults who did not seek treatment on medical advice belonged to the rural place of residence, during both the periods- 2014 and 2017–18. It was also found that majority of the adults with untreated ailments were either illiterates or had very low level of educational attainment (i.e. upto upper primary or below) and belonged to the bottom two wealth quartiles across both the rounds of NSS. An overwhelming majority of the ailing adults with unmet need for health care had no health insurance (76% and 83% in 71^st^ and 75^th^ rounds, respectively).

**Table 5:** Socio-economic differentials of unmet need for health care in adult population in India.

Covariates		2014 (71^st^ round)		2017–18 (75^th^ round)	
		Unmet need (%)	Chi-squared	Unmet need (%)	Chi-squared
Age	Younger Adults (20–44 years)	61.38	380000 Ϯ	58.53	330000 Ϯ
	Middle Aged (45–59 years)	38.62		41.47	
Sex	Male	42.05	31000 Ϯ	44.51	48000 Ϯ
	Female	57.95		55.49	
Place of Residence	Rural	70.69	500000 Ϯ	71.36	200000 Ϯ
	Urban	29.31		28.64	
Education	Illiterate	38.64	380000 Ϯ	34.97	120000 Ϯ
	Upper Primary or below	40.21		38.98	
	Secondary	9.16		9.64	
	Higher secondary or above	11.99		16.41	
Marital Status	Currently Married	19.43	31000 Ϯ	78.23	30000 Ϯ
	Others	80.57		21.77	
Religion	Hindu	81.91	290000 Ϯ	82.94	100000 Ϯ
	Muslim	12.16		11.94	
	Christian	2.82		2.65	
	Sikh	1.10		2.00	
	Others	2.01		0.48	
Social Group	ST	11.27	710000 Ϯ	9.18	230000 Ϯ
	SC	19.91		22.05	
	OBC	40.37		42.33	
	Others	28.44		26.44	
Economic Group	Poor	36.62	1200000 Ϯ	37.65	520000 Ϯ
	Lower Middle	28.29		25.26	
	Upper Middle	21.66		19.57	
	Rich	13.44		17.52	
Health Coverage	Not Covered	75.81	13000 Ϯ	82.61	240000 Ϯ
	Covered	24.19		17.39	
Household Size	4 or less	55.48	28000 Ϯ	57.62	8700 Ϯ
	5 to 6	30.48		27.02	
	7 or more	14.04		15.36	
Region	North	6.50	1300000 Ϯ	10.84	560000 Ϯ
	Central	16.69		24.15	
	East	33.61		31.74	
	Northeast	2.69		2.15	
	West	13.05		11.49	
	South	27.45		19.62	
**TOTAL**	** **	**100.00**	** **	**100.00**	** **

Note:

Ϯ p<0.001

Source: Authors’ calculation based on the NSS 71^st^ and 75^th^ rounds’ data on Social Consumption: Health

### Association between socio-economic factors and health seeking behavior

In this section, odds ratios are computed through logistic regression to examine the effect of the economic status (MPCE-based quartiles) on the health seeking behaviour of the ailing adult population in India, after controlling for other covariates (demographic, socio-economic, social support and geographical). The results of the logistic regression have been presented in Table 6.

**Table 6:** Association between socio-economic factors and health seeking behavior in adult population in India.

71^st^ round (2014)			75^th^ round (2017–18)	
Number of obs = 18,084		Pseudo R2 = 0.0492	Number of obs = 20, 148	Pseudo R2 = 0.0424
LR chi2(28) = 678.67		Log likelihood = -6554.50	LR chi2(25) = 524.69	Log likelihood = -5926.80
Prob > chi2 = 0.0000			Prob > chi2 = 0.000	
Dependent Variable			2014 (71^st^ round)	2017–18 (75^th^ round)
Medical Treatment Sought- Yes:1, No:0				
**Covariates**			Odds Ratio	Odds Ratio
Age	Middle Aged (45–59 years) ®			
	Younger Adults (20–44 years)		0.607 Ϯ	0.572 Ϯ
Sex	Female®			
	Male		0.916*	0.823 Ϯ
Place of Residence	Rural®			
	Urban		1.041	1.056
Education	Illiterate®			
	Upper Primary or below		1.532 Ϯ	1.284 Ϯ
	Secondary		1.596 Ϯ	1.327***
	Higher secondary or above		1.841 Ϯ	1.289***
Marital Status	Others®			
	Currently Married		1.051	1.120*
Religion	Muslim®			
	Hindu		0.798***	0.818***
	Christian		0.699***	0.645***
	Sikh		1.167	1.101
	Others		1.118	1.626**
Social Group	ST®			
	SC		1.412 Ϯ	1.710 Ϯ
	OBC		1.528 Ϯ	1.595 Ϯ
	Others		1.435 Ϯ	1.830 Ϯ
Economic Group	Poor®			
	Lower Middle		1.181**	1.161**
	Upper Middle		1.437 Ϯ	1.429 Ϯ
	Rich		1.850 Ϯ	1.588 Ϯ
Health Coverage	Not Covered®			
	Covered		0.909*	1.169**
Household Size	7 or more®			
	4 or less		0.725 Ϯ	0.921
	5 to 6		0.830***	0.959
Region	North®			
	Central		0.724***	0.999
	East		0.410 Ϯ	0.821**
	Northeast		0.325 Ϯ	0.513 Ϯ
	West		0.554 Ϯ	1.341***
	South		0.667 Ϯ	1.290***

Note: **®**Reference category

Ϯ p<0.001

*** p≤0.01

** p≤0.05

* p≤0.10

Source: Authors’ calculation from the NSS 71^st^ and 75^th^ rounds’ data on Social Consumption: Health

The results show that the economic status is a significant determinant of treatment seeking behavior of ailing persons. With reference to the poor, the lower middle, upper middle and rich, all have higher odds of seeking treatment for an aliment in both the rounds. Also, in both the rounds, ailing persons with a certain level of education have higher likelihood (odds) of seeking medical treatment than the illiterates.

Younger adults have lesser probability (odds) of seeking medical treatment than middle-aged population. Male ailing adults have lower odds of seeking treatment than their female counterparts. Urban dwellers have a higher probability (odds) of seeking treatment for a spell of ailment than their rural counterparts. Accessibility of health care services especially is an issue of concern in rural India [22, 66]. However, the association of place of residence with treatment seeking behavior was not statistically significant in our study. Those who are currently married have higher odds of treatment seeking than others (never married, widowed/ separated/ divorced). This is supported by studies that have found an adverse effect of widowhood on the health seeking behavior of women [67, 68]. In respect of religious groups, the odds of Hindu adult population seeking treatment for an illness are less likely than that of Muslim adults in both the rounds and the results are statistically significant at 01 percent level. This is not in line with the literature of socio-economic iniquities in health [46, 69]. In spite of the average socio-economic status of Indian Muslims being lower than their Hindu counterparts, Muslim advantage in certain health indicators (like child mortality) has been observed as a ‘paradox’ with very limited studies attempting to explain this phenomenon [70]. A study attempted to explain the Muslim advantage by suggesting that this may be an effect of the ‘omitted variables correlated with religion’ and ‘factors that operate through the community’ [71]. However, qualitative exploration of the unquantifiable cultural factors that overlap with religion in determining health seeking behavior is required to fully understand the Muslim advantage in this respect. SC, OBC and others have higher odds of seeking medical treatment than STs in both the rounds. This finding is in concurrence with studies that have found high prevalence of untreated morbidities among the Scheduled Tribes (STs) [72]. It has been argued that the lack of good quality and timely health care services at public facilities in tribal areas may be responsible for keeping STs away from availing medical treatment and causing them to resort to home-remedies, traditional healers, etc. [72, 73]. Adults covered by certain schemes for health expenditure support had lower odds of seeking treatment than those with no coverage in 71^st^ round although indicating a weaker association with the coefficient being significant at 08 percent level. However, in 75^th^ round, those with certain health insurance had higher odds of seeking treatment than those without any health coverage (significant at 1.2% level). This is supported by studies that have shown a strong association between health insurance coverage and treatment seeking behavior as financial cost acts as one of the major barriers to receiving health care [74, 75]. When medical costs have to be borne out-of-pocket in the absence of reimbursement provisions supported by health insurance coverage, ailments are likely to remain untreated. In case of household size, in the 71^st^ round, those living in a household of 4 or less or those living in households of size 5–6 had lesser odds of utilizing health care services with reference to household size of 7 or more. This is in contrast to studies that found under-utilization of health care services by individuals with large family sizes [76, 77]. This may be because a greater household size indicates a greater number of earning members and therefore the affordability of health care is better. However, the composition of the household in terms of earning members, dependent members (child and elderly), etc. needs to be investigated to come to a concrete conclusion in this respect. Household size didn’t, however, show a significant association with treatment seeking behavior in the 75^th^ round.

With respect to the regional dimension, adults living in central, east, west, northeast and south regions had lesser odds of utilizing health care compared to those living in northern India in 71^st^ round. However, in 75^th^ round, adults in western and southern regions showed higher odds of seeking medical treatment than those in North. Given that India witnesses varied levels of government spending on health care in its constituent states, the regional factor has strong associations with utilisation of health care by the poor [78].

### Socio-economic inequity in health care utilization: Concentration Index (CI)

**Fig 1:**
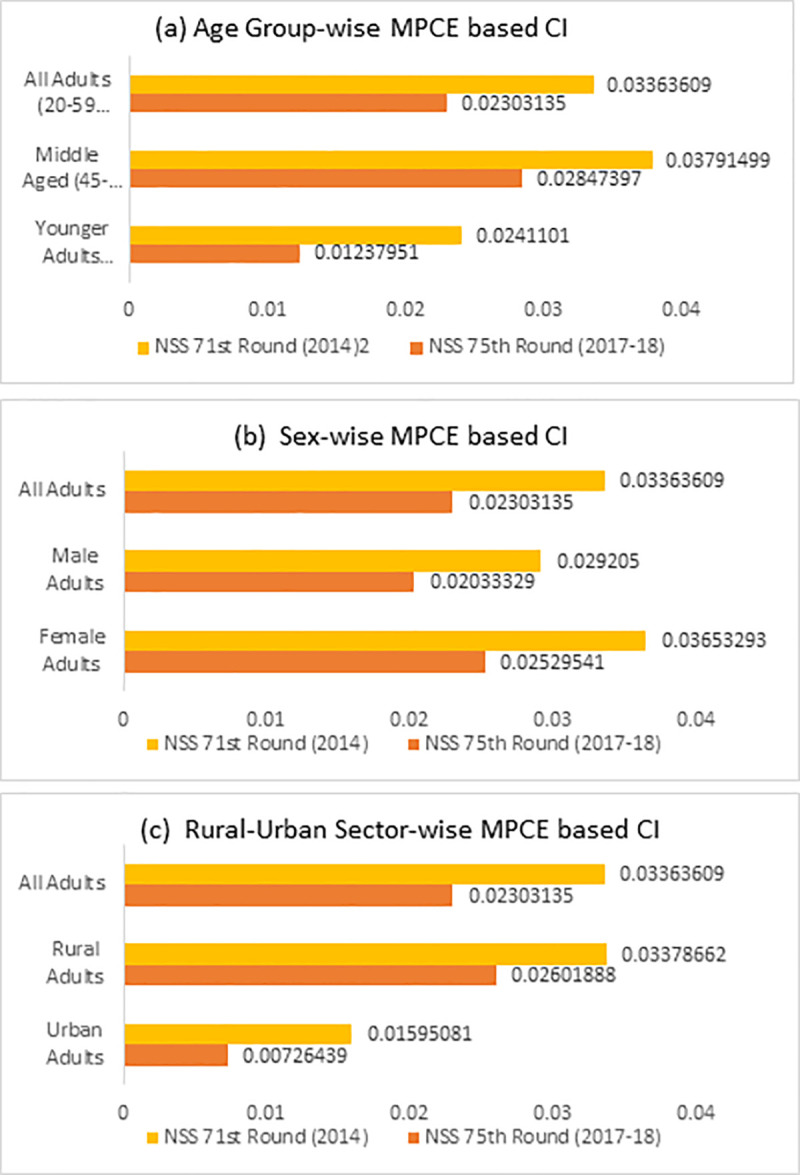
MPCE based Concentration Index (CI) for health care utilization among adult population in India, (a) Age group, (b) Sex and (c) Sector wise. Source: Authors’ calculation from the NSS 71^st^ and 75^th^ rounds’ data on Social Consumption: Health.

The results of the logistic regression established significant association between a person’s MPCE based economic status and their health seeking behavior, indicating the prevalence of socio-economic inequality in health care utilization for any spell of illness. In this section, we attempt to quantify the socio-economic inequalities in utilization of health care among the adult population by using Concentration Indices (CIs). Fig 1(A) shows the values of CIs of health care utilization for the entire adult population (20–59 years) and also separately for the younger adult population of 20–44 years and middle-aged population of 45–59 years, for both the NSS rounds (71^st^ and 75^th^). The results clearly show that the values of concentration indices in both the rounds, for all three categories of the adult population are positive, indicating that the distribution of health care utilization is pro-rich, demonstrating the phenomenon of elite capture. This means that, those who belonged to the poorest socio-economic group are in a more disadvantageous position than those who belonged to the richest quartile in terms of health care utilization.

The value of CI in health care utilization for the entire adult population (20–59 years) was 0.0336309 in 71^st^ round and 0.02303135 in 75^th^ round, indicating the existence of socio-economic inequalities in the utilization of health care among the adult population in India. Although the distribution of health care utilization still remains inequitably skewed in favor of the rich, the magnitude of inequity has shrunk over the period of time between 2014 (71^st^ round) and 2017–18 (75^th^ round). Moreover, the comparison of CI values among the younger adult population of 20–44 years and middle-aged population of 45–59 years, showed that, the degree of socio-economic inequality is greater among the middle-aged population than younger adults in both the rounds. Also, the magnitude of socio-economic inequality is found to be greater among adult females when compared to adult males and those adults residing in rural areas when compared to those in urban areas in both the rounds (Fig 1B and 1C).

### Major contributory factors in socio-economic inequity in health care utilization: Decomposition analysis

Although the values of CI reveal the existence and magnitude of socio-economic inequity, the CIs do not shed any light on the pathway through which the inequity occurs. Thus, the decomposition of concentration index is important to explore the major contributory factors that lead to socio-economic inequities in health care utilization. The results of decomposition analyses of health care utilization are presented in Table 7, for the entire adult population (aged between 20 and 59 years) in India and separately for the younger adults (20–44 years) and middle-aged adults (45–59 years). The analysis is focused on the relative contributions of each covariate to the overall inequality in health care service utilization of the adult population.

**Table 7:** Contribution of predictors to socio-economic inequity in health care utilization among adult population in India: Results of decomposition analysis.

Covariates	Adult Population (20–59 years)							
	2014 (71st round)				2017–18 (75th round)			
	Elasticity	CI	Absolute Contribution to CI	% Contribution	Elasticity	CI	Absolute Contribution to CI	% Contribution
Age Group 45–59 years	0.038	0.088	0.0034	10.030	0.034	0.051	0.0017	7.47
Female	0.030	-0.004	-0.0001	-0.390	0.020	-0.017	-0.0004	-1.50
Rural	-0.020	-0.199	0.0041	12.140	-0.015	-0.231	0.0036	15.44
Illiterate	-0.020	-0.259	0.0052	15.440	-0.010	-0.260	0.0025	10.74
Never married/ widowed/ divorced	-0.005	0.037	-0.0002	-0.520	-0.004	0.017	-0.0001	-0.27
Muslim	0.003	-0.135	-0.0004	-1.310	0.007	-0.101	-0.0007	-3.18
SC/ ST	-0.019	-0.171	0.0032	9.690	-0.004	-0.244	0.0010	4.45
Poor	-0.012	-0.862	0.0103	30.660	-0.014	-0.742	0.0103	44.67
No health coverage	-0.006	-0.058	0.0004	1.060	-0.036	-0.054	0.0020	8.47
Household size 7+	0.009	-0.345	-0.0032	-9.400	0.001	-0.286	-0.0002	-0.77
Northern Region	0.012	0.119	0.0014	4.160	0.002	0.197	0.0004	1.53
		Residual	0.0095	28.44		Residual	0.0030	12.94
				100				100
Covariates	Younger Adults (20–44 years)							
	2014 (71st round)				2017–18 (75th round)			
	Elasticity	CI	Absolute Contribution to CI	% Contribution	Elasticity	CI	Absolute Contribution to CI	% Contribution
Female	0.029	-0.003	-0.001	-0.310	0.015	-0.031	-0.0005	-3.64
Rural	-0.020	-0.185	0.004	15.160	-0.014	-0.232	0.0033	26.27
Illiterate	-0.024	-0.288	0.007	29.010	-0.008	-0.280	0.0023	18.19
Never married/ widowed/ divorced	-0.010	0.084	-0.001	-3.440	-0.008	0.103	-0.0008	-6.27
Muslim	0.005	-0.117	-0.001	-2.210	0.015	-0.044	-0.0007	-5.23
SC/ ST	-0.011	-0.143	0.002	6.270	0.004	-0.190	-0.0007	-5.36
Poor	-0.009	-0.839	0.007	30.800	-0.002	-0.750	0.0015	12.22
No health coverage	-0.031	-0.048	0.001	6.090	-0.054	-0.055	0.0030	23.80
Household size 7+	0.011	-0.334	-0.004	-15.110	0.000	-0.252	0.0001	0.69
Northern Region	0.016	0.101	0.002	6.530	0.006	0.218	0.0014	11.26
		Residual	0.007	27.21		Residual	0.0035	28.09
				100				100
Covariates	Middle Aged (45–59 years)							
	2014 (71st round)				2017–18 (75th round)			
	Elasticity	CI	Absolute Contribution to CI	% Contribution	Elasticity	CI	Absolute Contribution to CI	% Contribution
Female	0.024	-0.001	0.0000	-0.090	0.019	-0.002	-0.00004	-0.14
Rural	-0.021	-0.211	0.0045	11.750	-0.020	-0.230	0.00452	15.75
Illiterate	-0.013	-0.277	0.0036	9.500	-0.009	-0.278	0.00238	8.29
Never married/ widowed/ divorced	-0.001	-0.015	0.0001	0.050	0.000	-0.067	-0.00003	-0.11
Muslim	0.003	-0.133	-0.0004	-1.000	0.002	-0.147	-0.00028	-0.98
SC/ ST	-0.028	-0.201	0.0056	14.740	-0.011	-0.290	0.00316	11.01
Poor	-0.014	-0.887	0.0127	33.600	-0.018	-0.749	0.01324	46.18
No health coverage	0.017	-0.062	-0.0011	-2.850	-0.025	-0.046	0.00117	4.07
Household size 7+	0.007	-0.366	-0.0027	-7.100	0.001	-0.315	-0.00041	-1.42
Northern Region	0.007	0.162	0.0011	2.930	-0.001	0.194	-0.00022	-0.78
		Residual	0.0146	38.47		Residual	0.00520	18.12
				100				100

Source: Authors’ calculation from the NSS 71^st^ and 75^th^ Round data on Social Consumption: Health.

The estimates of the percentage contribution of various socio-economic covariates to overall inequality in health care utilization in case of the adult population of 20–59 years, show that the poor economic status of the adult population alone contributed 30.66% of total explained inequalities in the 71^st^ round. The share has increased to 44.67% in 75^th^ round. Illiteracy and rural sector together explained roughly over one-fourth of the inequalities in health care utilization of ailing adult population of 20–59 years in both 71^st^ (27.58%) and 75^th^ (26.18%) rounds. However, the contribution of illiteracy saw a decline by 5% from 71^st^ round to 75^th^ round, while the share of rural sector increased by 3% between 71^st^ and 75^th^ rounds. The contribution of ‘no health coverage’ to the total explained inequities has observed a sharp rise from 1.06% to 8.47% between the two rounds, while the contribution of SC/ST social group has declined from 9.69% to 4.45%. Although the distribution of Muslim religion across the economic groups is concentrated among the poor (CI = -0.135), there is little sensitivity of health care utilization to variation in this factor (represented by elasticity = 0.003), which is why this particular covariate makes very little contribution to the inequity of health care utilization. The eleven selected variables together explained 71.66% and 87.06% of total estimated inequalities in the 71^st^ and 75^th^ rounds respectively. The contribution of some factors like female sex, marital status other than currently married, Muslim religion, household size of 7 and above show a negative share of contribution. This is because the contribution of each factor is a product of elasticity and CI of that factor. If either of these parameters observe a negative value, the resultant product is a negative number and thus, the contribution is in negative terms.

The age-disaggregated decomposition analyses of health care utilization for the younger adults and middle-aged populations also bring some insightful results. In case of the younger adults, while the relative contribution of poor economic status alone contributed 30.80% of total explained inequalities in the 71^st^ round, this share declined drastically to 12.22% in the 75^th^ round. The contribution of ‘no health coverage’ has sharply increased from 6.09% to 23.80% across the two rounds for this age-group. The residual estimates remain roughly the same in both the rounds. In case of the middle-aged population, the relative contribution of poor economic status rose from 33.60% of total explained inequalities in 71^st^ round to 46.18% in the 75^th^ round. The contribution of rural place of residence has also increased from 11.75% to 15.75% across the two rounds. However, the contribution of SC/ ST social group has seen a decline from 14.74% to 11.01% between the 71^st^ and the 75^th^ rounds for the middle-aged adults. The residual estimates (38.47% in 71^st^ round and 18.12% in 75^th^ round) show that the eleven selected variables together explained a greater part of the total estimated inequalities in 75^th^ round in comparison to the 71^st^ round. The contribution of poor economic status to the total explained inequalities is more in case of middle-aged population compared to the younger adults in both the rounds.

## Discussion and conclusion

This paper made an attempt to contribute to the existing pool of literature in the domain of health inequity in India, by quantifying and decomposing the socio-economic related inequality in the health care utilisation, among adults (younger adults and middle-aged), by analyzing the two rounds of NSS- 71^st^ and 75^th^, corresponding to the Schedule number 25.0 (Social Consumption: Health). A number of intriguing findings are highlighted in this study. The results of logistic regression revealed that economic status continues to show a strong association with the treatment seeking behavior among the adults in India (with a statistical significance of 1%). In addition, the other important covariates—education, social group, and household size, have statistically significant effect on the variation in treatment seeking. These are important parameters for socio-economic gradient, so the statistical significance of these variables further substantiates the core contention of our proposition. The positive estimates of CIs across the two rounds of NSS suggested considerable socio-economic inequality. Health care utilization of the adults continues to be concentrated within higher socio-economic status, although, the magnitude of inequity in health care utilization has shrunk from 0.0336 in 2014 to 0.0230 in 2017–18. However, the relative contribution of poor economic status to the overall explained inequities in health care utilisation observed a rise in its share from 31% in 2014 to 45% in 2017–18. This resonates with the finding of a study [79], highlighting the fact that, even though economic status-related inequality in in-patient health care utilization has lowered, it hasn’t made the situation more equitable for the poor due to the factors like poor provisioning of public health facilities, increased out of pocket expenditure for treatment in private facilities, among others.

The findings of this study are in tune with previous studies on health inequities pertaining to both developed and developing countries, demonstrating the fact that the distribution of health care utilization is highly skewed, disfavoring the poor [11, 20, 22, 23, 25, 57, 80, 81]. Thus, the findings of this study affirm the significance of the socio-economic gradient theory. In India, a country with a very low human development index [82] and widespread impoverishment [26], the socio-economic gradient theory is a fitting explanation of inequities in health care utilization. The poor are highly constrained by multiple deprivations including impoverishment, malnutrition, lack of education, poor sanitation, unhygienic living conditions, etc. that increase their susceptibility to diseases. Poor income base coupled with lack of social security support and high opportunity costs of seeking treatment due to informal job contracts, substantially restrict the health seeking behavior of the poor [83, 84].

A study on health care inequities in Northern Indian states, found poor utilization of health care services by lower income groups [20]. The magnitude of inequity for both out-patient and in-patient health care utilization was pro-rich while the inequity was higher in rural areas compared to urban areas in majority of the Indian states [25]. A study on the need-standardized income-related horizontal inequity in health care utilization found that the elderly with poor economic status have greater unmet needs for health care service utilization due to the distribution of health care access being pro-rich [22]. Another study that investigated the horizontal inequity in health care utilization (out-patient care) found that the inequity in out-patient care was pro-rich for adult population groups [23]. Further, the distribution of hospital admission was found to be pro-rich [24]. Studies in support of the socio-economic gradient theory are not confined to India only but have been conducted across the globe. A study conducted in rural Bangladesh found that the socio-economic status overrides age and gender in determining the health seeking behavior of adults and elderlies [85]. A study based in Switzerland found that equity in access to health care particularly with respect to specialist visits, showed a pro-rich distribution [80]. An investigation into the socio-economic inequities in the health care utilization in South-America found high levels of pro-rich inequity [81]. Even though the level of utilization of the poor is lower than the richer economic groups, it is more likely that the poor spend more on health care as a share of income than the better-off and the majority of the spending are made out of pocket due to lack of health insurance coverage [4, 32].

As per NHSRC (2017) [33], an average Indian bore 70 per cent of the total medical expenses from their own pocket. Such a high catastrophic out-of-pocket expenditure on health care creates a huge burden on households belonging to even middle-income wealth quintiles that may be forced to slip into poverty due to unforeseen health shocks [32, 86–88]. To meet the expenses borne out-of-pocket, poorer households often resort to distress health financing. Such a situation widens the inequality across income categories and breeds conditions of perpetual poverty for the disadvantaged groups. The other side of this disease burden is the adverse effect of the income-generating capacities of the poor [30].

India, with a staggering disease burden, is facing multiple challenges to counter it due to regional imbalance and steep socio-economic gradient in health care accessibility. The widespread presence of socio-economic inequity is socially unjust, unethical and detrimental to human wellbeing. It is, therefore, imperative to reduce the socio-economic inequities and address this problem both from the demand as well as supply sides. Public health care infrastructure in India is critically malnourished due to persistent under-investments for a long period, with a sharp rural-urban divide. Public health care services even when available are perceived to be of poor quality [27] and often ailing persons have to resort to utilizing private health care services putting a huge burden on their expenses [89]. In order to ensure equitable health care accessibility to the poor, revamping the public health infrastructure is the foremost necessary condition from the supply side. Therefore, it is warranted that India ramps up investments and raises the budgetary allocation in the health care infrastructure and human resources, much beyond the current spending of 1.28% of its GDP as public expenditure on health [90]. Further, in order to reduce the existing socio-economic inequities from the demand side, there is an inherent need to strengthen the redistributive mechanisms by tightening the various social security networks, through efficient targeting and broadening the outreach capacity to the various vulnerable and marginalized socio-economic groups. Lack of awareness is another major constraint in treatment seeking behavior of the population and is grossly tied with the socio-economic gradient. Education is an important instrument which enables individuals to process information. Hence, demand for self-protection (in terms of both mitigating and averting behavior) from various morbidity risks can be effectively triggered by ensuring equitable access to education and health care facilities.

It is to be noted here that the present study suffers from the general limitations of subjectivity of perception and reporting bias, which are inherent to any self-reported data (in this case, the National Sample Survey data). Morbidities may have been under-reported by the poor due to their increased tolerance and lack of awareness of diseases. On the other hand, the self-reported data of whether or not any medical treatment was sought may have been over-reported due to the absence of a mechanism for cross-checking the reported information through medical prescriptions/ entry in public medical registers, diagnostic records, etc.
